# Oil-free hyaluronic acid matrix for serial femtosecond crystallography

**DOI:** 10.1038/srep24484

**Published:** 2016-04-18

**Authors:** Michihiro Sugahara, Changyong Song, Mamoru Suzuki, Tetsuya Masuda, Shigeyuki Inoue, Takanori Nakane, Fumiaki Yumoto, Eriko Nango, Rie Tanaka, Kensuke Tono, Yasumasa Joti, Takashi Kameshima, Takaki Hatsui, Makina Yabashi, Osamu Nureki, Keiji Numata, So Iwata

**Affiliations:** 1 RIKEN SPring-8 Center, Kouto, Sayo-cho, Sayo-gun, Hyogo 679-5148, Japan; 2 Department of Physics, POSTECH, Pohang 790-784, Korea; 3 Institute for Protein Research, Osaka University, Yamadaoka, Suita, Osaka 565-0871, Japan; 4 Division of Food Science and Biotechnology, Graduate School of Agriculture, Kyoto University, Gokasho, Uji, Kyoto 611-0011, Japan; 5 Department of Cell Biology and Anatomy, Graduate School of Medicine, The University of Tokyo, Hongo, Bunkyo-ku, Tokyo, 113-0033, Japan; 6 Department of Biological Sciences, Graduate School of Science, The University of Tokyo, Hongo, Bunkyo-ku, Tokyo 113-0033, Japan; 7 Structural Biology Research Center, KEK High Energy Accelerator Research Organization, Tsukuba, Ibaraki 305-0801, Japan; 8 Japan Synchrotron Radiation Research Institute, Kouto, Sayo-cho, Sayo-gun, Hyogo 679-5198, Japan; 9 Enzyme Research Team, Biomass Engineering Research Division, RIKEN Center for Sustainable Resource Science, Hirosawa, Wako-shi, Saitama 351-0198, Japan; 10 Department of Cell Biology, Graduate School of Medicine, Kyoto University, Yoshidakonoe-cho, Sakyo-ku, Kyoto, 606-8501, Japan

## Abstract

The grease matrix was originally introduced as a microcrystal-carrier for serial femtosecond crystallography and has been expanded to applications for various types of proteins, including membrane proteins. However, the grease-based matrix has limited application for oil-sensitive proteins. Here we introduce a grease-free, water-based hyaluronic acid matrix. Applications for proteinase K and lysozyme proteins were able to produce electron density maps at 2.3-Å resolution.

Using femtosecond X-ray pulses, serial femtosecond crystallography (SFX) offers a route to overcome radiation damage to small protein crystals via the “diffraction-before-destruction” approach^1^,^2^,^3^,^4^,^5^,^6^. This has expanded the window to obtain room temperature structures of proteins^7^,^8^,^9^,^10^,^11^,^12^,^13^. Liquid jet injection of small protein crystals is often exploited for the serial sample loading^14^. Continuous flow at relative high speed (~10 m/sec) of the liquid-jet injectors consumes 10~100 mg of the sample (5~6 hours/a full data set), which may not be ideal for X-ray free-electron lasers (XFELs) with low repetition rates (30~120 Hz at current facilities). Micro-extrusion of specimens using viscous media such as the lipidic cubic phase (LCP)^12^, grease^13^, and Vaseline (petroleum jelly)^15^ can maintain a stable stream at a low flow rate of 0.02~0.5 μl/min, which helps to reduce sample consumption. On the other hand, the viscous media tends to produce stronger X-ray scatterings that increase noise levels. Efforts to discover a sample delivery medium with lower background scattering has been ongoing, and recent demonstrations using agarose media are notable in that it has lower background scattering compared to the LCP media^16^. However, in general, viscous media tends to cause cracking and dissolution of protein crystals due to various physical or chemical events such as osmotic shock arising from the properties of viscous media.

Here we introduce a crystal carrier of water-based hyaluronic acid which is a good alternative to the grease matrix, to expand its application to grease-sensitive proteins. A highly viscous hydrophilic polymer was chosen from commercially available polymers. The practicality of the hyaluronic acid matrix as a protein carrier for SFX was demonstrated using two different types of proteins. The relatively low background scattering was notable in comparison to the grease matrix.

## Results & Discussion

### Data collection and structure determination

**Figure 1:**
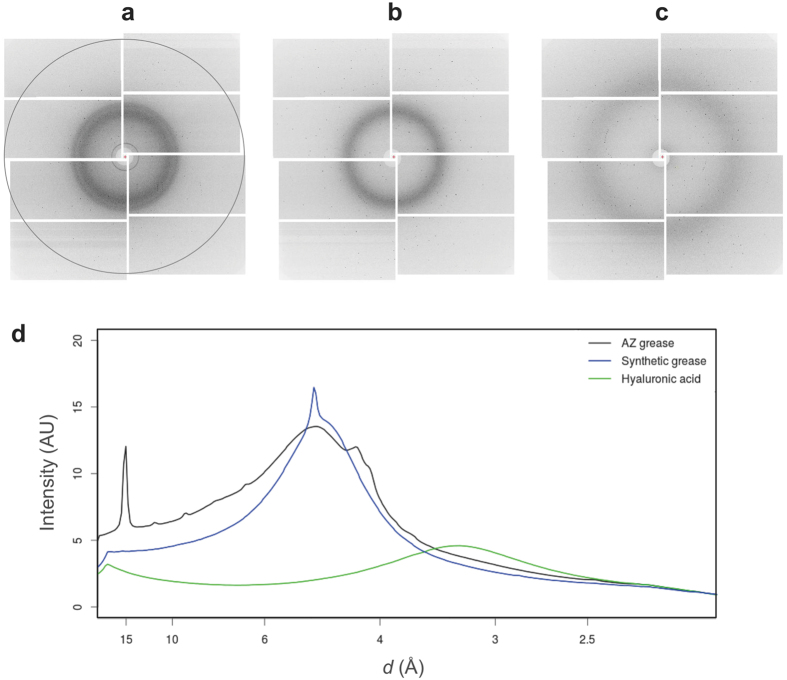
Typical XFEL single diffraction patterns from three carriers. (**a**) Mineral oil-based AZ grease, (**b**) Super Lube synthetic grease, and (**c**) hyaluronic acid. Resolution at the edges corresponds to ~2.3 Å (dashed circle). (**d**) The average background scattering intensities of ~2,000 images from each matrix. AZ grease, Super Lube synthetic grease and hyaluronic acid are depicted by the black, blue and green lines, respectively.

We carried out SFX experiments using femtosecond X-ray pulses from the SPring-8 Angstrom Compact Free Electron Laser (SACLA)^6^. The X-ray wavelength was kept at 1.77 Å (7 keV). At first, the background scatterings from three crystal matrix carriers, a mineral oil-based AZ grease^13^, a synthetic grease Super Lube^17^,^18^ and a hyaluronic acid aqueous solution, were compared. Grease generated diffuse scatterings in the resolution range of 4–5 Å (Fig. 1a,b). AZ grease gave a diffraction ring at ~14-Å resolution. For the Super Lube grease, we observed a diffraction ring at ~4.8-Å resolution in about 30% of all diffraction images (Supplementary Fig. 1). Weaker background scattering was noted when using hyaluronic acid compared with those of greases (Fig. 1c,d). In this study, using two carriers, synthetic grease Super Lube and hyaluronic acid matrices, we investigated proteinase K (5–10 μm) and lysozyme (7–10 μm) crystals (Supplementary Fig. 2) to demonstrate their general applicability as crystal carriers. A flow rate of 0.48 μl/min was used for all samples. The grease and hyaluronic acid matrices formed a stable flow for all protein samples (Supplementary Fig. 3).

**Figure 2:**
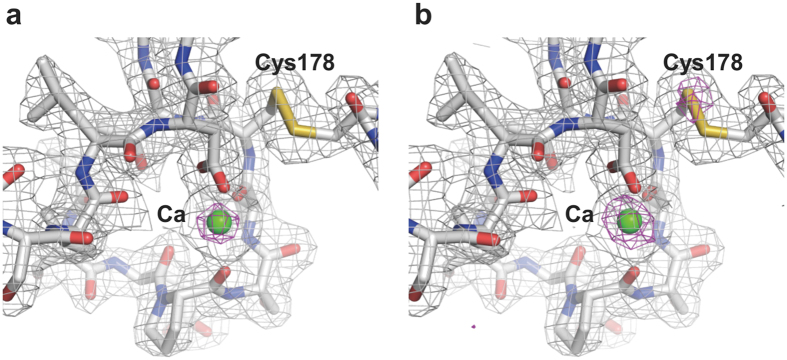
Electron density maps of proteinase K. Close-up views of the proteinase K structures for (**a**) Super Lube synthetic grease (PDB: 5B1D) and (**b**) hyaluronic acid (PDB: 5B1E) with 2*F*_o_ − *F*_c_ electron density maps contoured at the 1.0σ level (coloured gray) and the anomalous difference Fourier maps contoured at the 3.5σ level (coloured magenta). Bound calcium ion is depicted as a green sphere. These figures were drawn with PyMol (http://www.pymol.org).

With the SACLA running at a 30 Hz repetition rate, we were able to collect ~100,000 diffraction patterns in approximately 1 hour. In total about 30 μl of the sample volume was used with the crystal number density of 6.7 × 10^7^ crystals/ml (Table 1). We successfully indexed and integrated 21,000–27,000 patterns for each of the proteinase K and lysozyme crystals (space group *P*4_3_2_1_2). The crystals yielded data sets at 2.3-Å resolution with a completeness of 100% and an *R*_split_^19^ ranging from 8.5% to 9.7%. We determined and refined the crystal structures of proteinase K (Protein Data Bank (PDB) ID: 5B1D for grease and 5B1E for hyaluronic acid) and lysozyme (5B1F for grease and 5B1G for hyaluronic acid) at 2.3-Å resolution. Clear electron density maps of proteinase K and lysozyme were able to be observed (examples are shown here for proteinase K, Fig. 2).

### Sample preparation

Using the two matrix carriers, Super Lube grease and hyaluronic acid, we successfully collected the data sets for proteinase K and lysozyme at 2.3-Å resolution (Table 1). A 12% (*w*/*v*) hyaluronic acid matrix prepared by mixing 24% (*w*/*v*) hyaluronic acid aqueous solution with an equal volume of the supernatant solution in the crystal suspension solution was used for the lysozyme crystals. Optimizing the hyaluronic acid solution buffer is important to prevent any damage to the crystals. We have observed that before adding protein crystals it is essential to mix the hyaluronic acid aqueous solution with the supernatant solution or the crystal harvest solution, which helps to avoid osmotic shock to the crystals when mixing with the medium. The hyaluronic acid solution was saturated with the supernatant solution or the crystal harvest solution, and then protein crystals were added, which helps to avoid potential osmotic shock to the crystals. The unit-cell axes of the lysozyme crystals for the grease matrix were slightly shorter than those for the hyaluronic acid matrix (Table 1). Dehydration of protein crystals might have been induced during the sample preparation process of the water-free grease matrix. In such cases, a water-based medium can be helpful for preventing the contraction of the unit cell in the SFX experiments.

In comparison with LCP^12^, grease^13^ and Vaseline (petroleum jelly)^15^, water-based media such as hyaluronic acid and agarose^16^ produce lower background scattering noise; however, the agarose medium requires heat treatment at temperatures higher than 80 °C. The sample preparation in our technique can be performed by simply mixing with hyaluronic acid medium. In SFX, the grease matrix may not always be useful, because some proteins are damaged while being mixed and soaked in them. In the matrix technique using viscous media, the first step is to find a carrier for the protein crystals of interest that is suitable for data collection at room temperature. For SFX experiments, it is important to provide a wide repertoire of carrier media for a wide variety of proteins. Currently we are studying other crystal matrix carriers with low background scattering. For example, hydroxyethyl cellulose medium appears to be a good candidate.

### Background scattering & column diameter

The Super Lube grease tended to give a stronger background scattering in the resolution range of 4–5 Å than the hyaluronic acid (Fig. 1b,d). However, there was no noticeable difference in the data collection statistics or the electron density maps between the two carriers (Table 1 and Fig. 2). Statistics of *I*/*σ*(*I*) for proteinase K showed higher intensity values for the hyaluronic acid matrix at resolutions ranging from ~8 to ~3 Å in comparison to the grease matrix (Supplementary Fig. 4). However, it is reversed on the border of around 3 Å resolution, because water-based matrix gives a slightly higher background scattering in the resolution range of ~3.5–2.5 Å compared to the grease matrix (Fig. 1). To date, we have performed SFX experiments with the grease matrix using more than ten soluble proteins and three membrane proteins. However, we observed dissolution of crystals for one soluble and one membrane protein samples in the matrix. We do not yet have the data sets from these samples, but we confirmed that water-based matrix is useful for all these oil-sensitive crystals in SFX. These results suggest that the Super Lube grease has potential as a versatile matrix carrier, but the hyaluronic acid matrix would enable SFX experiments for grease-sensitive protein crystals.

Untreated Super Lube grease extruded through a 110-μm-i.d. needle tended to produce a larger-diameter grease column (approximately ~210 μm) about the size of the outer diameter (o.d.) of the needle, and similar to the mineral oil-based AZ grease^13^. By grinding the Super Lube grease for 30–60 min, the grease produced a sample column diameter of ~110 μm (Supplementary Fig. 2a). On the other hand, the hyaluronic acid matrix was extruded as a continuous column with a diameter of 110–130 μm through a 110-μm-i.d. needle (Supplementary Fig. 2b). A sample column with a smaller diameter contributes to reduce sample consumption and background noise from the matrices.

Recently, using the grease matrix technique, Yamashita and coworkers have demonstrated a single isomorphous replacement with anomalous scattering (SIRAS) phasing for Hg-derivatized luciferin-regenerating enzyme^17^. In addition, we have successfully determined the structure of native lysozyme with single-wavelength anomalous diffraction (SAD) by utilizing the anomalous signal of sulfur and chlorine^18^. One of the major challenges for phasing in SFX is to improve the signal-to-noise ratio. In this study, we could observe a weak anomalous scattering signal from the calcium atom in the proteinase K structures (Fig. 2). The anomalous difference Fourier maps showed that the signal from the calcium atom is stronger with the hyaluronic acid matrix than with the grease matrix. Furthermore, in the crystal structure for the hyaluronic acid matrix, we could observe anomalous signal from sulfur atoms (e.g. the sulfur atom of Cys178, Fig. 2b), which was not discernible when using the grease matrix. This technique using the matrices with low background scattering noise will contribute significantly to measuring weak anomalous signals for *de novo* phasing from SFX data.

In summary, using the hyaluronic acid matrix as a general carrier of protein microcrystals for serial sample loading in SFX, we successfully obtained the room-temperature structures at 2.3-Å resolution of two proteins in 5–10 μm microcrystals using less than 1 mg of sample. Oil- and water-based crystal carriers are complementary and their application to a wide variety of proteins is essential to firmly establish SFX. Recently, using viscous carrier media, synchrotron-based serial crystallography data collection at room temperature has also been demonstrated^15^,^20^. In the immediate future, the sample loading technique with a viscous medium which helps to reduce sample consumption will become more important in serial millisecond crystallography using synchrotron radiation. SFX has provided new opportunities for time-resolved studies of light-driven structural changes and chemical dynamics^21^,^22^,^23^. Matrix carriers with a stable sample flow and small diameter sample column should be applicable for time-resolved studies using pump-probe techniques.

## Methods

### Sample preparation

Proteinase K from *Engyodontium album* (No. P2308, Sigma) was crystalized by mixing a 1:1 ratio of 40 mg/ml protein solution in 20 mM MES–NaOH (pH 6.5) and a precipitant solution composed of 0.5 M NaNO_3_, 0.1 M CaCl_2_, 0.1 M MES–NaOH (pH 6.5). Microcrystals were produced by incubation for 5–10 min at 18 °C. A 1.0-ml sample of crystallization solution was centrifuged at 20 °C and 3,000 *g* for 3 min, and then the supernatant solution was removed. The crystals of proteinase K were suspended in 1.0 ml of the crystallization reagent. The crystal suspensions were filtered through a mesh (pore size, 30 μm). Lysozyme crystals were prepared as described previously^13^. Proteinase K and lysozyme samples were adjusted to a number density of 6.7 × 10^7 ^crystals/ml. The samples were then stored at 18 °C for proteinase K and 4 °C for lysozyme.

Synthetic grease Super Lube (No. 21030, Synco Chemical Co.) was ground using a mortar for 30–60 min. The crystals were mixed with the ground grease using the same procedure reported by Sugahara *et al.*^13^. For hyaluronic acid (No. H5388, Sigma), protein microcrystals were prepared according to the following procedures. After a 100-μl sample of storage solution was centrifuged for 10 sec, a 90-μl aliquot of supernatant solution was removed. For proteinase K crystals, an 8.0-μl aliquot of the crystal solution was dispensed into 72 μl of 12% (*w*/*v*) hyaluronic acid aqueous solution on a glass slide and then mixed with a spatula. For lysozyme crystals, a hyaluronic acid matrix was prepared by mixing a 1:1 ratio of 24% (*w*/*v*) hyaluronic acid aqueous solution and the supernatant solution of crystallization solution. An 8.0-μl aliquot of the crystal solution was dispensed into 72 μl of the hyaluronic acid solution. An aliquot (30 μl) of the sample was extruded into a 100-μl syringe (No. 1710, Hamilton).

### Data collection

We carried out the experiments using femtosecond X-ray pulses from the SPring-8 Angstrom Compact Free Electron Laser (SACLA)^6^. The X-ray wavelength was kept at 1.77 Å (7 keV) with a pulse energy of ~ 200 μJ. Each X-ray pulse delivered ~ 7 × 10^10^ photons within a 10-fs duration (FWHM) to the samples with a matrix. Data were collected using focused X-ray beams of 1.5 × 1.5 μm^2^ by Kirkpatrick-Baez mirrors^24^. The crystals in the matrix were serially loaded using a syringe injector installed in a helium ambiance, diffraction chamber. The experiments were carried out using a Diverse Application Platform for Hard X-ray Diffraction in SACLA (DAPHNIS)^25^ at BL3^26^. The microcrystals embedded in grease or hyaluronic acid matrix were kept at a temperature of approximately 20 °C. The sample chamber was kept at a temperature of ~ 26 °C and humidity greater than 80%. Each matrix with randomly oriented crystals was extruded through a syringe needle with an inner diameter (i.d.) of 110 μm (outer diameter (o.d.), 210 μm; No. 7803-05, Hamilton). The sample flow rate was 0.48 μl/min. Diffraction patterns were collected using a custom-built multiport CCD^27^.

### Background intensity determination

The background intensity was determined by a procedure similar to that used in^16^. First, the average (*m*) and standard deviation (*s*) of each detector pixel over images were calculated. To remove intensity contributions due to Bragg spots from protein crystals, pixels brighter than *m* + 3*s* were rejected. Remaining pixels were averaged again to yield a “clean” background image. This image was radially averaged by the resolution calculated from the detector metrology. When plotting Fig. 1d, datasets were scaled so that values at the highest resolution shell became the same.

### Structure determination

Diffraction patterns were processed by Cheetah^28^ adapted for the SACLA data acquisition system^29^. Each pattern with more than 20 spots was accepted as a hit, and indexed and integrated using CrystFEL^19^. Diffraction peak positions were determined using the built-in Zaefferer algorithm and passed on to DirAx^30^ for indexing. Monte Carlo integrated intensities from CrystFEL were converted to MTZ format. The structures were determined by the molecular replacement method using Molrep^31^ with search models (PDB: 5AVJ for proteinase K and 3WUL for lysozyme). Manual model revision was performed using Coot^32^. The program Phenix^33^ was used for structure refinement. Details of the data collection and refinement statistics are summarized in Table 1.

## Additional Information

**Accession codes:** The coordinates and structure factors have been deposited in the Protein Data Bank under the accession code 5B1D and 5B1E for proteinase K and 5B1F and 5B1G for lysozyme. Diffraction images have been deposited to CXIDB under ID #33 (lysozyme in grease) and #41 (others).

**How to cite this article**: Sugahara, M. *et al.* Oil-free hyaluronic acid matrix for serial femtosecond crystallography. *Sci. Rep.* **6**, 24484; doi: 10.1038/srep24484 (2016).

## Supplementary Material

Supplementary Information

## Figures and Tables

**Table 1:** Crystallographic statistics.

Protein	Proteinase K		Lysozyme	
Carriers	Super Lube	Hyaluronic acid	Super Lube	Hyaluronic acid
Data collection				
Space group	*P*4_3_2_1_2	*P*4_3_2_1_2	*P*4_3_2_1_2	*P*4_3_2_1_2
Unit-cell parameter				
*a* (Å)	68.8	68.9	79.1	80.3
*b* (Å)	68.8	68.9	79.1	80.3
*c* (Å)	109.1	109.4	38.0	38.7
Number of collected images	99, 912	99, 283	99, 774	104, 999
Number of indexed patterns	21, 480	21, 750	24, 652	27, 168
Indexing rate (%)§	21.5	21.9	24.7	25.9
Number of total reflections	4, 851, 553	4, 940, 994	2, 596, 103	3, 004, 570
Number of unique reflections	12, 196	12, 263	5, 727	5, 983
Resolution range (Å)	30.0–2.3 (2.34–2.30)	30.0–2.3 (2.34–2.30)	30.0–2.3 (2.34–2.30)	30.0–2.30 (2.34–2.30)
Completeness (%)	100 (100)	100 (100)	100 (100)	100 (100)
*R*_split_ (%)†	8.8 (10.7)	8.5 (11.2)	9.6 (10.5)	9.7 (13.3)
CC_1/2_ (%)	98.7 (97.0)	98.5 (95.8)	98.1 (96.9)	98.3 (95.2)
<*I*/*σ*(*I*)>	12.3 (8.9)	12.4 (8.7)	11.1 (10.4)	9.8 (7.6)
Wilson B (Å^2^)	35.6	36.1	37.5	39.1
Refinement				
*R*/*R*_free_ (%)	14.1/18.3	14.1/18.1	17.3/21.5	18.1/21.1
R.m.s. deviations				
Bond lengths (Å)	0.010	0.009	0.009	0.009
Bond angles (°)	1.073	1.055	1.135	1.162
PDB code	5B1D	5B1E	5B1F	5B1G

Values in parentheses are for the outermost shell. ^§^Percentage of images that were indexed.

^†^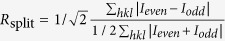.
