# Supplementary material for: Oil-free hyaluronic acid matrix for serial femtosecond crystallography
Source: Sci Rep. 2016 Apr 18;6:24484. doi: 10.1038/srep24484 (PMC4834484; doi:10.1038/srep24484)
Supplement: Supplementary Information [file srep24484-s1.pdf]

Oil-free hyaluronic acid matrix for serial femtosecond crystallography

Michihiro Sugahara*, Changyong Song, Mamoru Suzuki, Tetsuya Masuda, Shigeyuki Inoue, Takanori Nakane, Fumiaki Yumoto, Eriko Nango, Rie Tanaka, Kensuke Tono, Yasumasa Joti, Takashi Kameshima, Takaki Hatsui, Makina Yabashi, Osamu Nureki, Keiji Numata, So Iwata

Supplementary Figures

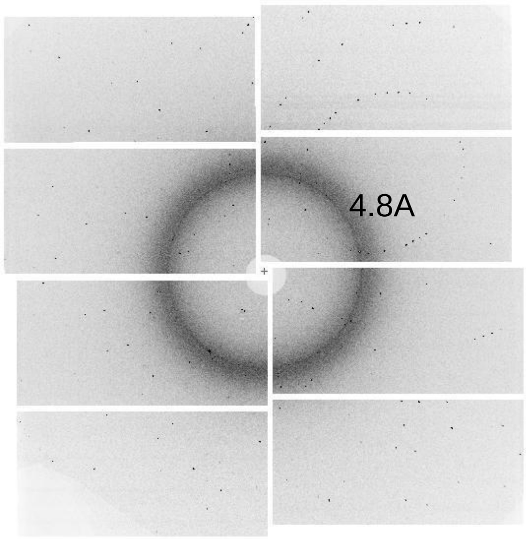

Supplementary Figure 1: A single diffraction pattern from Super Lube grease. Super Lube grease tended to give a diffraction ring pattern at ~4.8-Å resolution in ~30% of all diffraction images.

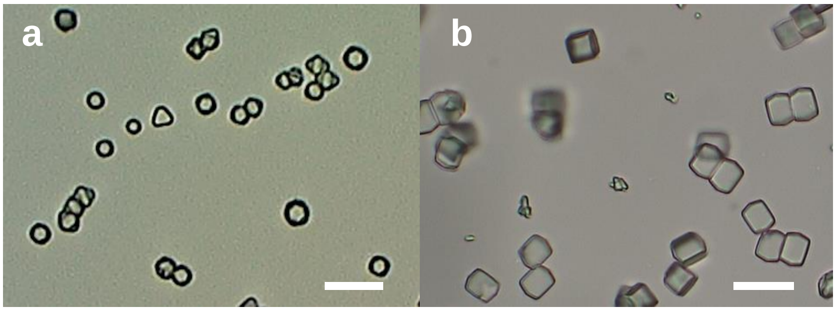

Supplementary Figure 2: Protein microcrystals used for SFX measurements. (a) Proteinase K, and (b) lysozyme crystals. Scale bars represent 20 μm.

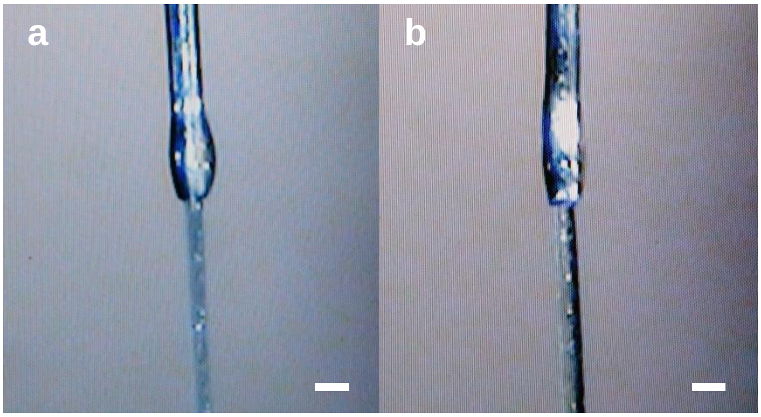

Supplementary Figure 3: Sample extrusion of the two crystal carriers through a 110-µm-i.d. needle at a flow rate of 0.48 µl/min. (a) Super Lube grease, and (b) hyaluronic acid were extruded as a continuous column to intersect with the XFEL beam. Scale bars represent 210 µm.

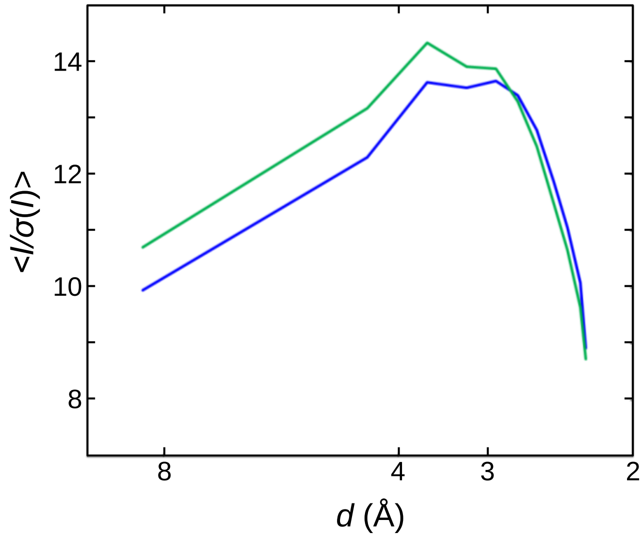

Supplementary Figure 4: Statistics of *I*/*σ*(*I*) for proteinase K protein. Two data sets of the grease and hyaluronic acid matrices are colored in blue and green, respectively.
